# Immunomodulatory Effects of *Anadenanthera colubrina* Bark Extract in Experimental Autoimmune Encephalomyelitis

**DOI:** 10.3390/cimb46080515

**Published:** 2024-08-10

**Authors:** Karla A. Ramos, Igor G. M. Soares, Larissa M. A. Oliveira, Mariana A. Braga, Pietra P. C. Soares, Gracimerio J. Guarneire, Elaine C. Scherrer, Fernando S. Silva, Nerilson M. Lima, Felipe A. La Porta, Teresinha de Jesus A. S. Andrade, Gagan Preet, Sandra B. R. Castro, Caio César S. Alves, Alessandra P. Carli

**Affiliations:** 1 Faculty of Medicine, Federal University of Jequitinhonha and Mucuri Valleys, Teófilo Otoni 39803-371, MG, Brazil; karlaaramos@yahoo.com.br (K.A.R.); igorgmsoares@yahoo.com.br (I.G.M.S.); larimaoliveira@yahoo.com.br (L.M.A.O.); marianaabraga1@yahoo.com.br (M.A.B.); pietrapcsoares1@yahoo.com.br (P.P.C.S.); gracimerio@yahoo.com.br (G.J.G.); caio_alves@ufvjm.edu.br (C.C.S.A.); alessandrapcarli@hotmail.com (A.P.C.); 2 Institute of Life Sciences, Federal University of Juiz de Fora, Governador Valadares 35010-177, MG, Brazil; elainecscherrer1@yahoo.com.br (E.C.S.); fernandossilva@yahoo.com.br (F.S.S.); bertelli_ribeiro@ufjf.edu.br (S.B.R.C.); 3 Department of Chemistry, Federal University of Goiás, Goiânia 74690-900, GO, Brazil; 4 Department of Chemistry, Federal University of Technology—Paraná, Londrina 86036-370, PR, Brazil; felipelaporta@utfpr.edu.br; 5 Nucleus of Applied Research to Sciences (NIAC), Federal Institute of Maranhão, Campus Presidente Dutra, Presidente Dutra 65760-000, MA, Brazil; teresinha.andrade@ifma.edu.br; 6 Marine Biodiscovery Centre, Department of Chemistry, University of Aberdeen, Aberdeen AB24 3UE, Scotland, UK; gagan.preet1@abdn.ac.uk

**Keywords:** immunomodulation, neurodegenerative disorders, multiple sclerosis, polyphenols, angico

## Abstract

This study aimed to evaluate the efficacy of the ethanolic extract of *Anadenanthera colubrina* in modulating the immune response in the Experimental Autoimmune Encephalomyelitis (EAE) model. The ethanolic extract of the dried bark was analyzed by ESI (+) Orbitrap-MS to obtain a metabolite profile, demonstrating a wide variety of polyphenols, such as flavonoids and phenolic acids. Various parameters were evaluated, such as clinical signs, cytokines, cellular profile, and histopathology in the central nervous system (CNS). The ethanolic extract of *A. colubrina* demonstrated significant positive effects attenuating the clinical signs and pathological processes associated with EAE. The beneficial effects of the extract treatment were evidenced by reduced levels of pro-inflammatory cytokines, such as IL1β, IL-6, IL-12, TNF, IFN-γ, and a notable decrease in several cell profiles, including CD8+, CD4+, CD4+IFN-γ, CD4+IL-17+, CD11c+MHC-II+, CD11+CD80+, and CD11+CD86+ in the CNS. In addition, histological analysis revealed fewer inflammatory infiltrates and demyelination sites in the spinal cord of mice treated with the extract compared to the control model group. These results showed, for the first time, that the ethanolic extract of *A. colubrina* exerts a modulatory effect on inflammatory processes, improving clinical signs in EAE, in the acute phase of the disease, which could be further explored as a possible therapeutic alternative.

## 1. Introduction

Multiple sclerosis (MS) is a chronic autoimmune and neurodegenerative disease marked by pathological inflammation, involving focal lymphocyte infiltration, which leads to axonal demyelination and neuronal cell death [1,2]. MS exhibits a significant gender disparity, occurring more frequently in women, which contributes to its irregular global distribution [3]. This disease is characterized by the infiltration of mononuclear cells, demyelination of axons, and gliosis in the myelin sheath. This process triggers the formation of multiple plaques in the central nervous system (CNS), resulting in a range of symptoms that can progress to total paralysis [4]. The treatment of MS can be complex and stands out for the high rate of adverse reactions, directly affecting the quality of life of patients, and the high cost [5,6]. Currently, a definitive cure for MS remains elusive.

Current mainstream treatments for multiple sclerosis often come with side effects that limit their widespread use. Additionally, some side effects, such as increased risk of lymphopenia, neutropenia, and infection, require careful monitoring to prevent serious toxicities [7]. Considering the numerous side effects caused by synthetic drugs currently employed in the treatment of multiple sclerosis (MS) and the increasing evidence that natural compounds can treat MS effectively with minimal side effects, there is a pressing need to explore new natural alternative medicines in the pursuit of effective MS treatments [8]. In this context, plant-derived products have been extensively investigated as a foundation for numerous treatments targeting various diseases, both through traditional practices and the extraction of bioactive compounds from plant species [9]. Among the plant species with recognized therapeutic properties for mitigating inflammatory processes, and which may serve as an important source of phytocompounds with anti-inflammatory action for conditions such as MS, is *Anadenanthera colubrina* (Fabaceae).

*Anadenanthera colubrina* (Vell.) Brenan, commonly known as “Angico”, is a representative species of the Brazilian flora and is highly recognized for its medicinal properties and widespread adoption within the population [10,11]. It belongs to the angiosperm family Fabaceae, which boasts the highest diversity and species count in the Brazilian flora, with 795 genera and nearly 20,000 species, constituting 50% of the country’s endemic plants [12,13]. The genus *Anadenanthera* comprises two to four species, with *A. colubrina* as one of them [14,15]. Widely distributed across various Brazilian regions, except Rio Grande do Sul, Santa Catarina, Espírito Santo, Alagoas, and the entire northern region, this typical woody species is often found in the Brazilian Caatinga biome [5,14].

Its leaves and bark are harvested, dried, and processed for medicinal purposes through various methods [16]. Due to its extensive use in traditional medicine and commercial applications (e.g., termite-resistant wood, civil engineering, and household objects), it has gained recognition as an essential medicinal plant in conservation efforts [17].

As is well-known, “Angico” is used as a traditional remedy for various diseases, including flu, nasal congestion, cough, sore throat, stomach pain, diarrhea, and lung problems, and as an expectorant and antiseptic. Hence, the leaves or aerial parts of the plant find application in addressing conditions like anemia, inflammation, cancer, and various other health concerns [10,18,19]. Also, the bark of “Angico” is commonly used by the northeastern population of Brazil in various forms such as teas, infusions, decoctions, tinctures, and syrups to address conditions such as gonorrhea, asthma, bronchial disease, sore throat, cough, uterine problems, rheumatism, inflammation, and ovarian infection [20,21]. Ethnopharmacological studies delving into the traditional use of “Angico” have highlighted its effectiveness in addressing inflammatory issues [22].

Several studies on *A. colubrina* have demonstrated its promising therapeutic properties in various aspects. It has shown effectiveness in addressing inflammation at the general level [23], exhibiting notable antifungal and antiproliferative properties [24], and demonstrating anti-inflammatory and antioxidant effects [25,26]. Additionally, it has also exhibited anti-HIV activity probably due the reduction of the Tat function, resulting in down regulation of Env expression, and the presence of catechin and gallic acid, who act as HIV integrase and reverse transcriptase inhibitors, as evidenced by the research conducted by Maia et al. in 2022 [27]. This growing body of evidence underscores the diverse therapeutic applications of *A. colubrina* and accentuates its potential significance in developing novel treatments across a spectrum of medical conditions.

The phytochemical analysis of *A. colubrina* reveals that the extract derived from its bark contains flavonoids, phenols, saponins, steroids, tannins, triterpenes, xanthones, phenolic acids, fatty acids, and sugars [28,29]. The phenolic compounds found in the bark of *A. colubrina* are valued for their bioactive properties, which contribute to their therapeutic potential [28,30]. Moreover, flavonoids possess antioxidant and anti-inflammatory properties, which justify their utilization in treating various diseases with an inflammatory component, such as neurodegenerative conditions [31].

The present study aims to evaluate the pharmacological effect of the ethanolic extract from *A. colubrina* barks in modulating the immune response in the Experimental Autoimmune Encephalomyelitis (EAE) model. Notably, it has been known that this EAE model holds significant importance in comprehending the pathogenesis of MS as well as designing therapeutic interventions [32,33,34]. Our findings indicate that the ethanolic extract derived from *A. colubrina* bark exhibits a high concentration of flavonoids and phenolic acids, rendering it valuable for treating inflammatory conditions. To date, this is the first report showing the application of *A. colubrina* extract in the treatment of EAE in the acute phase of the disease.

## 2. Materials and Methods

### 2.1. Plant Material

The plant material was collected in the Alto Mucuri Environmental Protection Area in March 2021, in the community of Baixão, municipality of Caraí-MG (lat: 17.277828 long: 41.6555 WGS84). After being sanitized, the samples were placed in an oven at 40 °C for 2 h and stored at room temperature. Subsequently, botanical identification was performed by morphological analysis and comparison to Brazilian and worldwide virtual collections of the Reflora 2021 Virtual Herbarium and the New York Botanical Garden 2021. One specimen from *Anadenanthera colubrina* was prepared and deposited at the Jeanine Felfili Dendrological Herbarium (HDJF) from the Federal University of the Jequitinhonha and Mucuri Valleys as voucher HDJF 3355. The material was registered in SisGen under number A5C1263. For the preparation of the extract, the barks of *A. colubrina* were carefully removed, washed, dried, grounded in an industrial blender, and mixed with ethanol P.A., in the proportion of 100 g of the plant material in 400 mL of ethanol, homogenizing them and keeping in a closed container for 48 h. After 48 h, they were filtered under vacuum and stored in a freezer at −80 °C for 24 h. After this period, they were lyophilized and stored in a −20 °C freezer.

### 2.2. Animals and Ethical Aspects

C57BL/6 female mice, 6–8 weeks old, from the Federal University of Minas Gerais (UFMG) animal facility were used. The animals were randomly housed in appropriate polypropylene cages and kept at a constant temperature of 22–23 °C, with free access to feed and water, at the animal house of the Faculdade de Medicina do Mucuri—Universidade Federal dos Vales do Jequitinhonha e Mucuri (UFVJM) at the Mucuri Campus. All protocols were performed responsibly according to ethical principles regarding the use of animals in experiments, approved by the Ethics Committee on Animal Use (CEUA)-UFVJM (protocol No. 052021R).

### 2.3. Induction of Experimental Autoimmune Encephalomyelitis

C57BL/6 females were divided into 3 groups (n = 8): (a) EAE (induced and treated with PBS); (b) EtAc (induced and treated with ethanolic extract of *A. colubrina* barks—200 mg/kg/day); and (c) CN (control not induced and treated with PBS). In previous studies by our group, we determined that a dose of 200 mg/kg is the ideal dose for analysis. In order to deepen our knowledge of the potential uses of this plant, we are maintaining this concentration as the standard [25,26].

Each induced animal was immunized subcutaneously (s.c) with 100 µL, on each side of the dorsal region near the base of the tail, with 100 µg of MOG35-55 (GenOne) emulsified in complete Freund’s adjuvant (Sigma Chemical Co., St. Louis, MO, USA) in a 1:1 ratio, supplemented with 4 mg/mL of *Mycobacterium tuberculosis*—H37 RA (Difco). On the day of immunization and after 48 h, each animal received 0.3 µg of the pertussis toxin (Sigma) intraperitoneally (i.p.).

### 2.4. Treatment with Ethanolic Extract of Anadenanthera colubrina (EtAc) and Clinical Assessment

The treatment was initiated on the 15th day post-induction (dpi) of EAE by gavage and extended until the 21st dpi, considering the day of induction as day 0. The treatment was performed with 100 µL of PBS or 100 µL of EtAc (200 mg/kg) resuspended in PBS. Regarding the clinical evaluation, mice were weighed daily from the day of induction (day 0) until the day of euthanasia (day 21 after immunization). The animals were also assessed for neurological disability using the De Paula et al. (2008) [35] adapted scale.

On the 21st dpi, the mice were euthanized by lethal doses of xylazine (10 mg/Kg) and ketamine (100 mg/Kg). Blood (cardiac puncture) was collected to obtain plasma, and the spinal cord and brain were excised for cytokine concentrations and for evaluating cellular profile, histopathology, and demyelination.

### 2.5. Histopathological Analysis

The animals were euthanized and perfused with buffered formalin. After that, the spinal cord was excised and fixed in buffered formalin. Cross-sections of the spinal cord, at cervical, thoracic, and lumbar levels, were processed for paraffin perfusion. Sections of 5 µm were stained with hematoxylin and eosin (H&E) to assess the presence of inflammatory infiltrates. Sections of 8 µm were stained with Luxol fast blue to evaluate demyelination. The sections were analyzed in a double-blind analysis and documented on a Nikon microscope. The inflammatory index was calculated as the average number of perivascular inflammatory infiltrates in white matter lesions. The extent of myelin loss was calculated by calculating the ratio of the areas of myelin loss and the entire total white matter area using the Image J software (Version 1.54, National Institutes of Health, Bethesda, MD, USA).

### 2.6. Brain and Spinal Cord Cells Isolation and Evaluation of the Cellular Profile

Excised brains and spinal cords were macerated and filtered using a 70 µm cell strainer (BD Biosciences, Pharmigen, San Diego, CA, USA) in RPMI 1640 medium with 10% fetal bovine serum (FBS) (Sigma). Mononuclear cells were collected after a Percoll density gradient centrifugation and washed with staining buffer (PBS, 1% FBS, 0.09% sodium azide) [15]. The cell pellet was suspended in ACK solution, centrifuged at 350× *g* for 5 min, and suspended in staining buffer for flow cytometry analysis. Cells were counted in a Neubauer chamber and incubated with anti-mouse CD80-PerCP-Cy5.5, anti-mouse MHC-II-BB515, anti-mouse CD86-BV421, anti-mouse CD11c-PE-Cy7, anti-mouse CD4-PerCP-Cy5.5, and anti-mouse CD8-APC (BD Biosciences Pharmingen, San Diego, CA, USA) antibodies, as determined by the manufacturer. After 30 min of incubation at 4 °C, cells were washed with PBS, 1% SFB, and 0.09% sodium azide and fixation buffer containing paraformaldehyde (BD Biosciences, Pharmigen, San Diego, CA, USA) and washed in permeabilization buffer (BD Biosciences, Pharmigen, San Diego, CA, USA). After extracellular labelling, the cells were subjected to intracellular labelling with anti-mouse IFN-γ- BV421, anti-mouse IL-10-PE, anti-mouse IL-17A-PE-Cy7, and anti-mouse Foxp3-Alexa Fluor 488 (BD Biosciences Pharmigen, San Diego, CA, USA). Cell capture was performed using the FACSVerse flow cytometer (Becton Dickinson, Franklin Lakes, NJ, USA), and analyses were performed with FCS Express version 3.

### 2.7. Cytokine Content

To evaluate cytokine levels, portions of the organs (brain and spinal cord) were frozen at −80 °C and thawed only on the day of processing. After thawing, the organs were macerated in cytokine extraction buffer (100 mg/mL) composed of: 0.4 M NaCl, 0.05% tween 20, 0.5% bovine serum albumin-BSA, 0.1 M phenyl-methyl-sulfonyl fluoride-PMSF, 0.1 M benzethonium chloride-BC, 10 mM ethylene diamino tetra acetic acid-EDTA, and 20 pM aprotinin-AP. Then, the material was homogenized and centrifuged at 10,000 rpm for 15 min at 4 °C, and the supernatants were collected and frozen at −80 °C. Cytokine concentrations in the supernatants were determined by the ELISA method, using commercially available antibodies and concentrations according to the procedures recommended by the manufacturers for the cytokines sought. The amounts of cytokines were calculated from the standard curves obtained by different concentrations of the respective recombinants for IL-1β, IL-6, IL-10, IL-12, TNF, and IFN-γ (BD Biosciences).

### 2.8. Compound Profiling

The phytochemical extracts, at concentration of 300 µg mL^−1^, were analyzed through ultra-high-resolution mass spectrometry (UHRMS) analysis. The samples were directly infused into a Q-Exactive hybrid Quadrupole-Orbitrap mass spectrometer using an electrospray ionization source (ESI). The metabolite fingerprinting was obtained in positive ionization mode (ESI (+)). The conditions for the ESI source were as follows: spray voltage was set to 3.5 kV, the capillary temperature was 250 °C, the S-lens RF level was maintained at 60 V, the sheath gas flow rate was 45 L min^−1^, and the auxiliary gas flow rate was 10 L min^−1^. High-resolution mass spectra were acquired in Full MS/data-dependent MS2 (dd-MS2) mode for both ESI positive. The full MS scan range was *m/z* 100–1200. The five most abundant precursors (TopN 5, loop count 5) were sequentially selected for collision-induced dissociation. Collision energies for the target analytes were set at 20, 30, and 35 eV. The resolving power was 140,000 for full MS and 70,000 for dd-MS2 acquisitions.

The compound profiling of the *A. colubrina* bark extract was obtained through tandem mass spectrometry data in positive ionization mode (ESI (+) Orbitrap-MS) combined with molecular networking analysis and dereplication in silico tools. High-resolution mass spectra were acquired in Full MS/data-dependent MS2 (dd-MS2) mode, covering a mass range of *m*/*z* 100–1200 in the full MS scanning experiments [36]. Additionally, Junior et al. [26] previously determined the chemical profile.

### 2.9. Statistical Analysis

Statistical analyses were performed using GraphPad Prism version 5.0 software. The results were expressed as mean ± standard error. Numerical variables were evaluated in the different groups by the Kolmogorov–Smirnov normality test for the Gaussian distribution of the data. To determine significant differences between the groups of mice, the non-parametric Mann–Whitney test or two-way ANOVA with Dunnett’s correction were employed, when appropriate, with a significance level of less than 5% (*p* < 0.05).

## 3. Results and Discussion

### 3.1. EtAc Improves the Clinical Signs of Experimental Autoimmune Encephalomyelitis (EAE)

**Figure 1:**
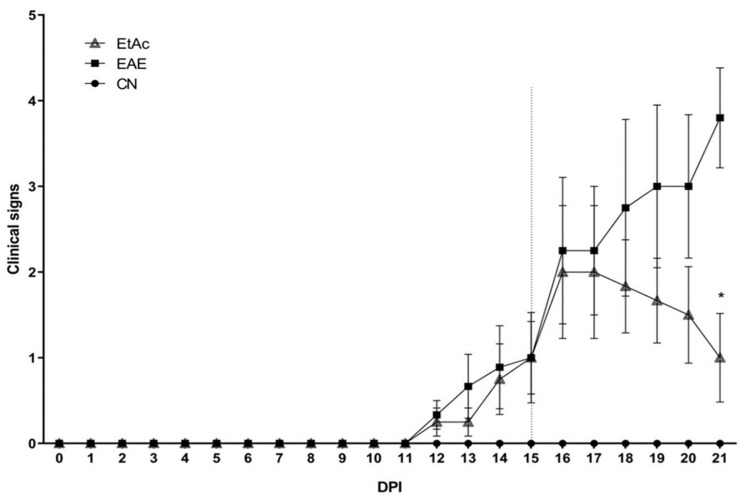
Clinical signs of EAE. Animals (n = 8/group) were monitored daily for clinical signs of EAE after immunization with 100 µg of MOG_35–55_ peptide. Mice were treated with 200 mg/kg of the ethanolic extract of *A. colubrina* barks (EtAc) for six days. The dotted line indicates the start of treatment. Each dot represents the arithmetic mean ± SEM. * indicates *p* < 0.05 compared to induced and PBS-treated animals (EAE), analyzed by two-way ANOVA with Dunnett’s correction. CN = negative control (not induced and treated with PBS).

It is established that the EAE model is used to assess neurological disability and the evolution of multiple sclerosis in mice. Distinctive indicators, including tail paralysis and hind limb weakness, are recognized manifestations of axonal damage in the EAE model in mice [35,37]. Figure 1 shows that the EAE group displayed characteristic signs of the disease, such as weakness/paralysis of the tail and hind limbs, becoming evident from day 12 and reaching a peak at day 21, confirming the successful induction of the disease. In contrast, the ethanolic extract from the *A. colubrina* (EtAc) group showed an increase in the clinical signs at day 16 post-immunization, maintaining a significantly lower clinical score than the EAE group until day 21 (*p* < 0.05). Hence, this observation indicates a reducing effect of the EtAc on the development and progression of clinical symptoms in the EAE model in the acute phase of the disease.

Flavonoids, which are secondary compounds abundantly present in *A. colubrina* [22], have gained increasing attention for their potential to ameliorate the clinical manifestations of experimental autoimmune encephalomyelitis (EAE) [38,39,40,41,42,43]. Therefore, it is reasonable to suggest that the observed effects on clinical signs following EtAc treatment could be attributed to the notable abundance of flavonoids in this extract detected through high-resolution mass spectrometry analysis; however, other studies should be conducted to figure if the flavonoids alone, or other constituents, improve the clinical signs of EAE. Notably, this study is the first report to demonstrate the effects of *Anadenanthera* sp. in this model.

### 3.2. Histological Evaluation of Inflammatory Infiltrates

**Figure 2:**
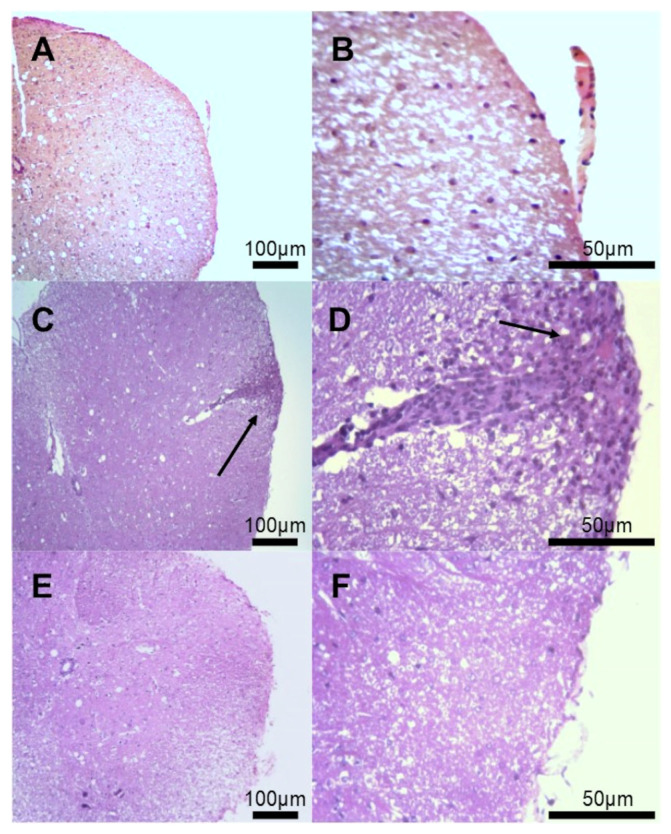
Histopathology of the spinal cord of mice. Histopathology of the spinal cord of mice immunized or not immunized with 100 µg of MOG_35–55_ (n = 8/group). Figures are representative of the histological analysis of each experimental group: CN= non-immunized and PBS-treated group (**A**,**B**), EAE = immunized and PBS-treated group (**C**,**D**), EtAc = immunized and treated with 200 mg/kg ethanolic extract of *A. colubrina* barks for six days (**E**,**F**). The examined groups representative sections (5 µm) were stained with hematoxylin and eosin (H&E) to analyze the cell infiltrate. Original magnification: 10× objective (**A**,**C**,**E**), 40× (**B**,**D**,**F**). Scale bars = 100 µm (10×) and 50 µm (40×). Arrows indicate cellular infiltrates.

Regarding the early stages of MS lesions, perivascular inflammatory infiltrates containing T and B cells exist near active tissue damage [44,45]. In analyzing histological sections in the spinal cord, the EAE group showed inflammatory infiltrates in the pachymeningeal and leptomeningeal sites (Figure 2C,D) compared to the non-immunized group (Figure 2A,B). Mice treated with EtAc showed fewer foci of inflammatory infiltration in the meningeal site (Figure 2E,F). In addition, the EtAc treatment reduced the inflammatory index in the spinal cord (2.16 ± 0.23 vs. 4.82 ± 0.27, *p* < 0.001) when compared to the EAE group. The histological slides of the non-immunized group (Figure 2A,B) were considered normal, without inflammatory infiltrates.

### 3.3. Histological Evaluation of Demyelination

**Figure 3:**
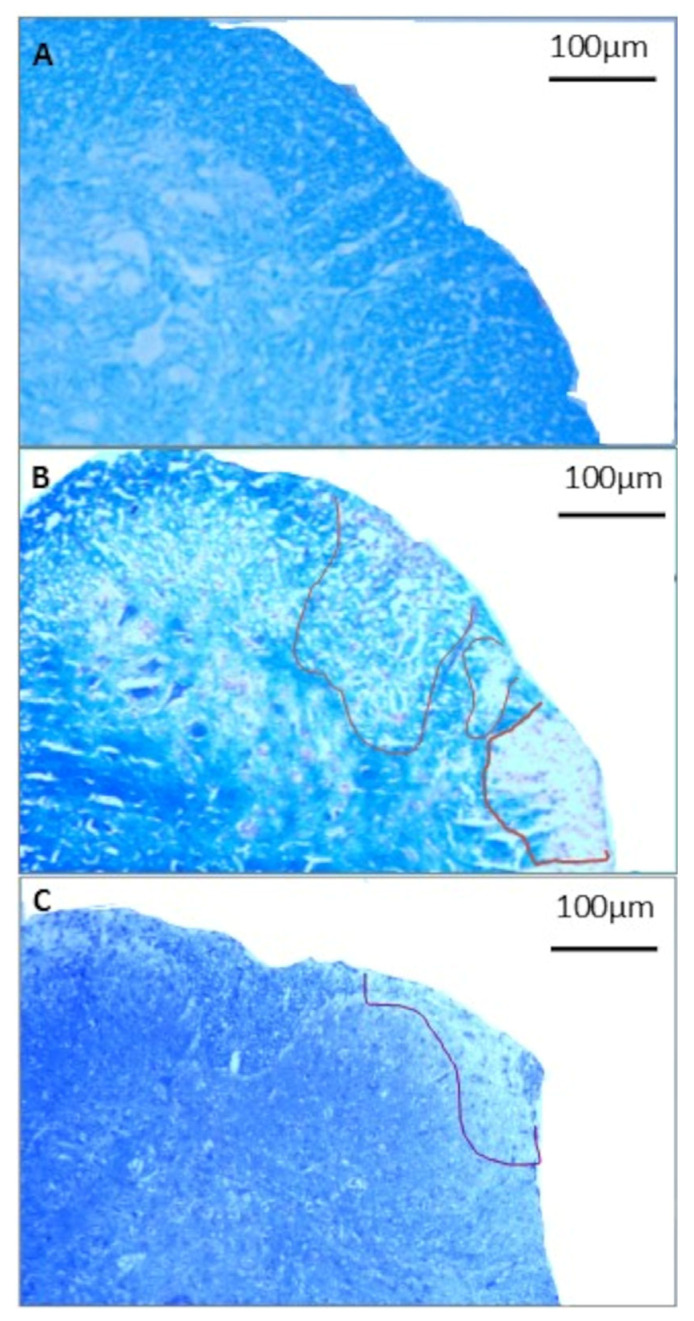
Demyelination of the spinal cord of mice. Histopathology of spinal cords of mice immunized or not immunized with 100 µg of MOG35–55 (n = 8/group). Figures are representative of the histological analysis of each experimental group: CN= non-immunized and PBS-treated group (**A**), EAE = immunized and PBS-treated group (**B**), EtAc = immunized and treated with 200 mg/kg ethanolic extract of *A. colubrina* barks for six days (**C**). Representative sections (8 µm) of the examined groups, stained with Luxol fast blue, for analysis of the demyelination. Original magnification: 10× objective. Scale bars = 100 µm. Delimited areas = areas of demyelination.

The examination of demyelination sites in the spinal cord of mice (Figure 3) provides valuable insights into the impact of EtAc treatment on the progression of EAE. The observed demyelination sites in the EAE group in the spinal cord slides (Figure 3B) indicate an active tissue injury process associated with vacuolization and secondary degeneration of parenchyma. In contrast, the EtAc group presents a noteworthy reduction in demyelination in the spinal cord slides (Figure 3C), with a general appearance similar to the histological slides of the non-immunized group (Figure 3A), suggesting a potential protective effect of *A. colubrina*, which could result in improved neurological outcomes. In addition, the EtAc treatment reduced the myelin loss index in the spinal cord (12.10 ± 2.33 vs. 24.75 ± 2.47, *p* < 0.001) when compared to the EAE group. Hence, the observed attenuation of demyelination relates to the earlier findings of reduced inflammatory infiltrates in the histological analysis. The correlation between decreased cellular infiltrate and reduced demyelination in the EtAc group underscores the potential of *A. colubrina* as a modulator of the inflammatory processes underlying EAE. However, further investigations are required to elucidate the precise mechanisms through which *A. colubrina* exerts its protective effects on demyelination, paving the way for potential therapeutic applications.

### 3.4. Expression of Cellular Markers in the Central Nervous System

**Figure 4:**
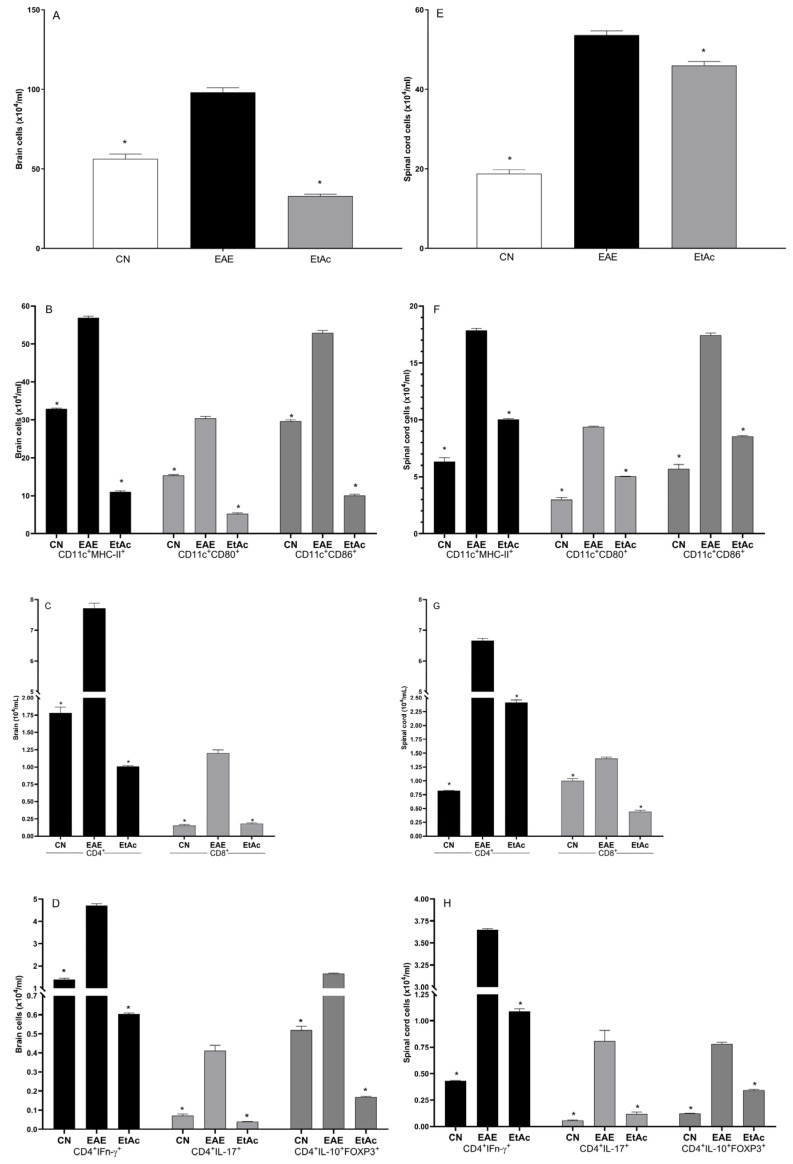
Cellular profile. Mononuclear cell counts (**A**,**E**) and cellular profile determination (**B**–**D**,**F**–**H**) in the brains (**A**–**D**) and spinal cords (**E**–**H**) of mice immunized or not immunized with 100 µg of MOG_35–55_ (n = 8/group). Mice were treated with 200 mg/kg of the ethanolic extract of *A. colubrina* barks (EtAc) for six days. Each bar represents the arithmetic mean ± SEM. * indicates *p* < 0.05 compared to induced and PBS-treated animals (EAE). CN = negative control (not induced and treated with PBS).

The analysis of the mononuclear cell counts and the cellular profile in the brain and the spinal cords are shown in Figure 4. The application of the EtAc treatment resulted in a notable decrease in the cell count within the brain and spinal cord. Furthermore, compared to the EAE group, the EtAc treatment exhibited a significant reduction across all cellular profiles examined (Figure 4). This reduction in cell numbers aligns with the previously noted mitigated inflammatory infiltrates and demyelination sites in the histological analysis, indicating a broader impact of EtAc on the immune response within the CNS.

Immune cells appear to mediate the critical neurodegenerative and inflammatory processes in the CNS of individuals with MS. In the CNS, cells such as infiltrating macrophages, microglia, B cells, and dendritic cells can express co-stimulatory molecules and class II MHC molecules that may be directly linked to the initiation and development of MS and EAE [46]. Notably, the co-stimulatory molecules CD80, CD86, and class II MHC molecules expressed by microglia are present in CNS lesions of EAE and MS [47]. Treatment with EtAc led to a reduction in the number of CD11c^+^MHC-II^+^, CD11^+^CD80^+^, and CD11^+^CD86^+^ cells in the brain and spinal cord, associated with the improvement of clinical signs and the results of the histopathological study with luxol fast blue. Specifically, the present study reveals a considerable decrease in demyelination areas in the mice, which was observed on day 21.

During EAE, the infiltrating dendritic cells (CD11c^+^) and macrophages into the CNS act as highly efficient antigen-presenting cells with greater efficiency when compared to resident CNS cells [48]. In addition to this, CD4^+^ T cells secreting IFN-γ (Th1) or IL-17 (Th17) have a leading role in the pathogenic lesions of EAE and MS, together with CD8^+^ T cells [49,50,51]. Treatment with EtAc led to a reduction in the number of CD8+, CD4+, CD4+IFN-γ, and CD4+IL-17+ in the spinal cords and brain, connecting the results observed in the clinical and histopathological scores of the animals.

Th17 cells represent an essential link to the inflammatory and neurodegenerative aspects found in MS through the secretion of cytokines, such as IL-17, that may damage the integrity of the blood–brain barrier (BHE) and consequently stimulate the migration of cells such as macrophages and neutrophils into the CNS [52]. On the other hand, IFN-γ is essential for Th1 cell-mediated immune responses and regulates T-cell expansion, activation, homeostasis, and survival [53]. IFN-γ is found in higher levels in the lesions of MS and EAE, which is associated with the inflammatory response mediated by IFN-γ-producing Th1 cells [54]. Hence, the observed alterations in cellular profiles and cytokine-secreting T cells following EtAc treatment underscore its high potential as a modulator of immune responses.

### 3.5. Cytokine Analyses

The levels of IL-1β, IL-6, IL-12p70, IFN-γ, TNF, and IL-10 in the supernatants of spinal cord and brain macerates were measured on day 21 post-immunization (Table 1 and Table 2).

In the brain macerates, the EtAc group showed lower levels of IL-12p70 and TNF and increased secretion of IL-10 compared to the EAE group (Table 1).

Treatment with EtAc significantly reduced the levels of all the dosed pro-inflammatory cytokines in the spinal cord macerates, except IL-6 and IL-12p70 (Table 2).

Studies have already shown that the *Anadenanthera* extracts can reduce inflammatory cytokines in vitro and, in vivo, the extracts can reduce the paw edema induced by carrageenan due to the increase in the IL-10 release in the paw [26,29]. This study showed that EtAc improved the clinical score of EAE, which may be correlated to the reduction in the levels of pro-inflammatory cytokines and the cellular profile.

### 3.6. Compound Profiling of Anadenanthera colubrina Bark Ethanolic Extract

In metabolomics research, the ESI (+) Orbitrap-MS-based compound profiling provided molecular insights into the chemical composition of bioactive compounds within the ethanolic extract from *A. colubrina* bark. The results unveiled a wide range of polyphenols, prominently featuring phenolic acids and flavonoids, renowned for their anti-inflammatory properties [55]. In this work, the untargeted compound profiling showed the presence of the compounds *p*-hydroxybenzoic acid, chlorogenic acid, ferulic acid, gallic acid, methyl gallate, *p*-coumaric acid, cinnamic acid, apigenin, catechin, epigallocatechin, hesperidin, hyperoside, isorhamnetin, myricetin, naringin, quercetin, and rutin, all previously identified in *A. colubrina* [22,26].

**Figure 5:**
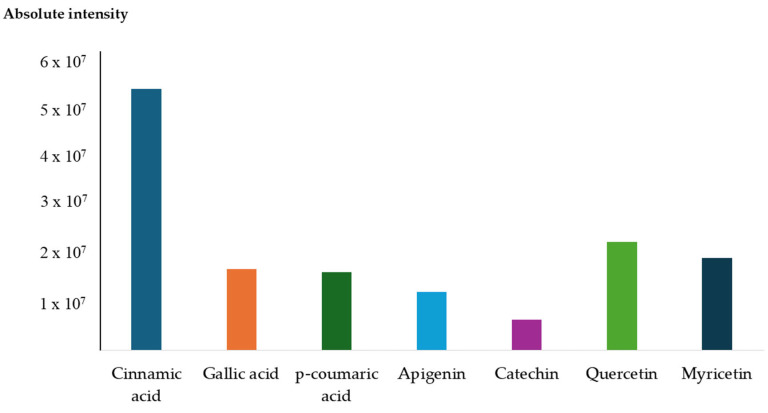
Absolute intensity of the most abundant phenolic acids (cinnamic acid, gallic acid, and *p*-coumaric acid) and flavonoids (apigenin, catechin, quercetin, and myricetin) annotated through ESI (+) Orbitrap-MS analysis of the ethanolic extract from *A. colubrina* bark.

The ethanolic extract of *A. colubrina* bark revealed significant levels of phenolic acids and flavonoids, particularly cinnamic acid and quercetin as the major compounds. Additionally, notable abundances of flavonoids such as apigenin, catechin, and myricetin and phenolic acids such as gallic acid and *p*-coumaric acid were also detected in high abundance (Figure 5). These phenolic compounds are believed to contribute to the observed bioactivity against EAE.

**Figure 6:**
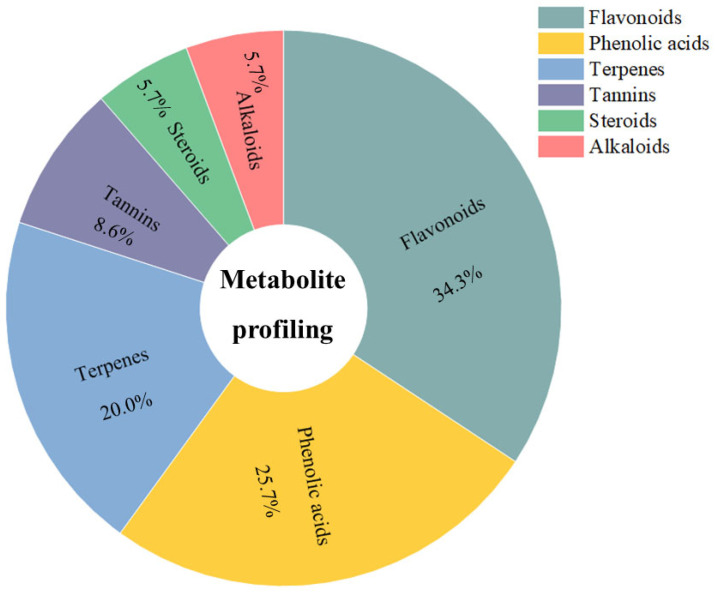
ESI (+) Orbitrap-MS-based metabolite profiling of *A. colubrina* showing the major classes identified in bark ethanolic extract.

Furthermore, utilizing the MolNetEnhancer tool [56] analysis enabled a more in-depth exploration of the chemical classes of secondary compounds predominantly identified in EtAc. Notably, flavonoids constituted a substantial proportion, accounting for 34.3% of the compounds. Phenolic acids followed closely at 25.7%, while terpenes, tannins, steroids, and alkaloids contributed 20%, 8.6%, 5.7%, and 5.7%, respectively, as shown in Figure 6. These findings highlight the rich phytochemical diversity present in EtAc, emphasizing its potential as a source of therapeutic compounds. The prevalence of flavonoids and phenolic acids, known for their anti-inflammatory and antioxidant properties, aligns with the observed positive effects of EtAc in mitigating clinical signs and modulating immune responses in the context of EAE. As the first report detailing the comprehensive secondary metabolite profile of *A. colubrina*, this study sets the stage for further exploration of the specific bioactive compounds contributing to its observed immunomodulatory effects and therapeutic potential.

## 4. Conclusions

These findings indicate that the ethanolic extract from *A. colubrina* bark possesses therapeutic potential for reducing clinical signs and pathological processes associated with EAE, in the acute phase of the disease, likely attributed to its high flavonoid and phenolic acids content, identified as major classes. A decrease in demyelination and inflammatory infiltrates in the spinal cord is observed through histopathological analysis, indicating a protective effect on the nervous tissue. The extract’s ability to modulate inflammatory processes, reduce cellular infiltration, and alleviate demyelination highlights its potential as a treatment option for EAE and warrants further investigation to elucidate the mechanism underlying the action of the extract and the isolated compounds and to assess the long-term safety and efficacy of this treatment. These findings aim to harness the therapeutic benefits of *A. colubrina*, offering a novel approach to address immune dysregulation and providing hope for developing effective treatments.

## Figures and Tables

**Table 1:** Evaluation of cytokines in the brain.

Cytokines	Groups Mean ± Standard Error			*p* * Value
	CN	EAE	EtAc	
IL-1β	1410 ± 60.55	1691± 27.05	2238± 67.89	0.02
IL-6	667.3 ± 9.10	691.5 ± 7.05	868 ± 55.16	0.016
IL-12p70	2397 ± 39.84	1543 ± 194.2	698.1 ± 45.95	0.005
IFN-γ	62.45 ± 6.84	91.75 ± 6.75	192.40 ± 11.68	0.028
TNF	484.0 ± 43.46	636.8 ± 17.55	435.5 ± 45.87	0.003
IL-10	3788 ± 158.5	4143 ± 194.8	5554 ± 319.4	0.004

Cytokine levels (pg/mL) in the supernatants of brain macerates of mice (n = 8/group) immunized with 100 µg of MOG_35–55_. Mice were treated with 200 mg/kg of *A. colubrina* barks (EtAc) ethanolic extract for 6 days. Data are presented as the arithmetic mean ± SEM. * compared to the induced and treated group with PBS (EAE). CN = negative control (not induced and treated with PBS).

**Table 2:** Cytokine evaluation in the spinal cord.

Cytokines	Groups Mean ± Standard Error			*p* * Value
	CN	EAE	EtAc	
IL-1β	1290 ± 46.95	1685± 69.05	1021± 36.78	0.008
IL-6	822.5 ± 35.57	1354 ± 160.5	805.4 ± 149.5	0.056
IL-12p70	3557 ± 212.9	4403 ± 497.7	2766 ± 775.4	0.191
IFN-γ	158.5 ± 10.70	232.5 ± 25.41	101.1 ± 3.59	0.007
TNF	1960 ± 20.57	2076 ± 67.64	1417 ± 83.48	0.008
IL-10	748.6 ± 74.69	300.6 ± 7.21	118.8 ± 24.60	0.007

Cytokine levels (pg/mL) in the supernatants of spinal cords macerates of mice (n = 8/group) immunized with 100 µg of _MOG35–55_. Mice were treated with 200 mg/kg of *A. colubrina* barks (EtAc) ethanolic extract for six days. Data are presented as the arithmetic mean ± SEM. * compared to the induced and treated group with PBS (EAE). CN = negative control (not induced and treated with PBS).

## Data Availability

Data are contained within the article. Further inquiries can be directed to the corresponding authors.
